# The role of PDIA3 in oral squamous cell carcinoma and its value as A diagnostic and prognostic biomarker

**DOI:** 10.1016/j.heliyon.2023.e22596

**Published:** 2023-11-22

**Authors:** Lin Wang, Xinxin Wang, Jia Zhang, Jiafeng Duan, Chengfang Tang, Linmei Zhang, Hui Zeng, Hantong Li, Yuefan Li, Yan Zhou

**Affiliations:** a College of Stomatology, Xi'an Medical University, Xi'an, Shaanxi, 710021, China; b Key Laboratory of Shaanxi Province for Craniofacial Precision Medicine Research, College of Stomatology, Xi'an Jiaotong University, Xi'an, Shaanxi, 710004, China; c Laboratory Center of Stomatology, College of Stomatology, Xi'an Jiaotong University, Xi'an, Shaanxi, 710004, China; d Department of Implant Dentistry, Xi'an Nobel Dental Hospital, Xi'an, Shaanxi, 710021, China

**Keywords:** OSCC, PDIA3, Biomarker, Diagnosis, Prognosis

## Abstract

**Background:**

This study aimed to investigate the role of protein disulfide isomerase A3 (PDIA3) in oral squamous cell carcinoma (OSCC) and evaluate its significance as a diagnostic and prognostic biomarker.

**Methods:**

Comprehensive bioinformatics analysis of the OSCC dataset from The Cancer Genome Atlas (TCGA) was performed. PDIA3 was depleted in CAL27 and SCC25 OSCC cells by transfection with PDIA3-specific siRNA oligos. The effects of PDIA3 downregulation on cell viability, apoptosis, and cell migration were evaluated using CCK8, ELISA, and wound healing assays, respectively.

**Results:**

The mRNA and protein expression of PDIA3 was significantly up-regulated in OSCC tissues compared to adjacent normal tissues. Knockdown of PDIA3 led to significantly decreased cell viability, increased apoptosis, and suppressed migratory ability in OSCC cells. The Kaplan-Meier survival curve showed that patients with higher PDIA3 expression levels had shorter survival than those with low PDIA3 levels. The receiver operating characteristic (ROC) curve indicated that PDIA3 had high sensitivity and accuracy for detecting OSCC (area under the curve (AUC): 0.917, CI: 0.879–0.955). Univariate and multivariate Cox regression analyses identified PDIA3 as an independent prognostic factor of OSCC. Furthermore, the depletion of PDIA3 inhibited AKT activity in OSCC cells. Gene set enrichment analysis (GSEA) indicated that PDIA3 is involved in various important biological functions and signaling pathways closely related to cancer development.

**Conclusion:**

PDIA3 plays an oncogenic role in OSCC and represents a good candidate as a diagnostic and prognostic biomarker for OSCC.

## 1 Introduction

Oral squamous cell carcinoma (OSCC) is a type of cancer that originates in the cells lining the oral cavity, including the tongue, lips, buccal mucosa, subgingival and supragingival tissues, and hard palate [1]. It is the most common type of oral cancer, accounting for approximately 90 % of all cases [2]. OSCC caused an estimated 300,400 new cases and 145,400 OSCC-related deaths in 2012 [3]. Approximately 80 % of patients with OSCC are infected with the high-risk human papillomavirus (HPV) 16 and 18 [4]. Treatment options for OSCC typically include surgery combined with chemotherapy, radiotherapy, or both. Despite improvements in the diagnosis and treatment of OSCC in recent years, the prognosis for advanced-stage OSCC remains poor, and the 5-year survival rate is below 50 % because of high recurrence and metastasis rates [5]. Therefore, identifying novel biomarkers and therapeutic targets is crucial for early diagnosis and effective treatment of OSCC.

Protein disulfide isomerase A3 (PDIA3), also known as ERp57, is an important member of the protein disulfide isomerase family [6]. PDIA3 is mainly localized in the endoplasmic reticulum. As a component of the molecular chaperone complex for calcium-binding and calcium mesh proteins, PDIA3 guides the folding of newly synthesized glycoproteins in the endoplasmic reticulum, which is important for the proper functioning of cells [6]. In recent years, several studies have indicated that PDIA3 plays an important role in tumorigenesis, cell proliferation, apoptosis, tumor metastasis, angiogenesis, and chemoresistance [7,8]. For example, in hepatocellular carcinoma, PDIA3 expression is associated with increased tumor cell proliferation and decreased apoptosis, and increased expression of PDIA3 indicates poor prognosis [9]. PDIA3 is also overexpressed in colon [10] and breast cancers [11] and is involved in the formation and development of these two types of cancers. PDIA3 downregulation inhibits the proliferation and invasion of human acute myeloid leukemia cells [12]. Furthermore, PDIA3 has been shown to promote radioresistance in laryngeal cancer cells by directly activating STAT3 signaling [13]. Given its role in cancer, PDIA3 has emerged as a potential biomarker and therapeutic target for cancer treatment. Notably, the role(s) of PDIA3 in OSCC remains largely unknown.

Therefore, we investigated the expression, function, and clinical relevance of PDIA3 in OSCC by performing comprehensive bioinformatic analyses and experimental studies. The goal of the present study was to understand the role(s) of PDIA3 in OSCC and evaluate its potential as a biomarker and therapeutic target in OSCC.

## 2 Materials and methods

### 2.1 Obtaining the cancer genome atlas (TCGA) data

The mRNA expression data of 335 OSCC cases and 31 normal tissue samples, along with clinical data including age, sex, pathological stage, and prognosis, were obtained from the TCGA database (https://portal.gdc.cancer.gov/). We compared the mRNA expression of PDIA3 in OSCC and normal tissues using the “Limma” package of the R software (version 3.6.3).

### 2.2 Protein expression of PDIA3 in the human protein atlas (HPA)

The protein expression of PDIA3 in normal and OSCC tissues was obtained from the HPA database (antibody number: HPA002645) (https://www.proteinatlas.org/) [14].

### 2.3 Evaluation of PDIA3 as a diagnostic and prognostic biomarker

The overall survival (OS), disease-specific survival (DSS), and progression-free interval (PFI) of patients with high or low PDIA3 expression were analyzed using the Kaplan-Meier survival analysis. We performed this analysis using the Kaplan-Meier survival “survival” package for survival analysis and the “survminer” package for visualization in the R software. The “pROC” package of R was used to calculate the area under the curve (AUC) values and plot the receiver operating characteristic (ROC) curves to evaluate the significance of PDIA3 as a diagnostic biomarker for OSCC. We also conducted univariate and multivariate Cox regression analyses to further evaluate the prognostic value of PDIA3 expression in OSCC.

### 2.4 Cell culture

CAL27 and SCC25 cell lines were obtained from the Institute of Oral and Maxillofacial Surgery, Nanchang University, and cultured in high-glucose DMEM (Gibco, USA) supplemented with 10 % fetal bovine serum (FBS) (Bioexplorer, USA) and 1 % penicillin/streptomycin (Solarbio, China) in a humidified atmosphere containing 5 % CO_2_ at 37 °C.

### 2.5 siRNA transfection

siRNA oligos specifically targeting PDIA3 were chemically synthesized by GenePharma (Shanghai, China). The sequences of si-PDIA3 were sense: 5′-GGAAUUGUCAGCCACUUGATT-3,’ antisense:5′-UCAAGUGGCUGACAAUUCCTT-3.' Non-targeting siRNA (siNC) was used as a control. The sequences of the control siRNAs were sense: 5′-UUCUCCGAACGUGUCACGUTT-3,' antisense:5′-ACGUGACACGUUCGGAGAATT-3.' The siRNA oligos were transiently transfected into cells using Advanced DNA RNA Transfection Reagent™ (ZETA LIFE Inc., USA) according to the manufacturer's instructions. Briefly, CAL27 and SCC25 cells were seeded in a 6-well plate. When the confluence reached 80 %, the cells were transfected with 12 μL siRNA (20 μM) and 12 μL transfection reagent, which were mixed at room temperature for 15 min. After 24 h, the culture medium was removed, and fresh DMEM was added to continue the culture.

### 2.6 CCK8 for cell viability

Cell proliferation was assessed using the cell counting kit-8 (CCK-8) (ZETA LIFE Inc., USA). Cells were transfected with siPDIA3 or control siRNA as described above. Dispense 100 μL of the cell suspension (1000 cells/well) in a 96-well plate and incubate the plate in an incubator. Cell viability was measured 24, 48, 72, and 96 h after plating the cells. Briefly, 10 μL CCK-8 solution was added to each well, the plate was incubated for 1 h in the incubator, and the absorbance was measured at 450 nm using a microplate reader.

### 2.7 Apoptosis assay by ELISA

Cell apoptosis was measured using the Cell Death Detection ELISA kit (Roche, USA), according to the manufacturer's instructions. In this assay, the level of cytosolic nucleosomes is used as an indicator of apoptosis in cells. After transfection, the cells were plated in 96-well plates at 1 × 10^4^ cells/well in triplicate. Apoptosis was evaluated 72 h after plating cells according to the manufacturer's protocol. The colorimetric change due to peroxidase conversion of the substrate was measured by measuring the absorbance at 402 nm using a plate reader.

### 2.8 Western blotting

Total protein was extracted using RIPA buffer supplemented with a protease inhibitor cocktail (NCM Biotech Co. Ltd., China) on ice. Protein concentration was determined using the BCA assay (Boster Biological Technology Co. Ltd, China). Cell lysates were denatured in sodium dodecyl sulfate (SDS) sample loading buffer at 95 °C for 10 min and separated on 10 % polyacrylamide gels. Proteins were then transferred to polyvinylidene fluoride (PVDF) membranes (Millipore, USA). Membranes were blocked with 5 % non-fat milk in TBST buffer for 1 h at room temperature and then incubated overnight at 4 °C with rabbit polyclonal anti-PDIA3 (1:2000, Proteintech, USA), rabbit polyclonal anti-AKT (1:2000, Proteintech, USA), rabbit anti-phospho-AKT (Ser473) (1:1000, Proteintech, USA), or rabbit polyclonal anti-β-actin (1:5000, Boster Biological Technology Co. Ltd, China). The membranes were then incubated with goat anti-rabbit secondary antibody (1:5000, Boster Biological Technology Co. Ltd., China) for 2 h at room temperature. Bands were visualized using enhanced chemiluminescence (ECL; NCM Biotech, USA), imaged, and quantified using a ChemiDoc Imaging System (Bio-Rad, USA).

### 2.9 Human sample collection

Tissue samples of oral squamous cell carcinoma (OSCC) and adjacent normal tissues were collected from patients diagnosed with OSCC at the Oral and Maxillofacial Surgery Department of the Stomatological Hospital of Xi'an Jiaotong University. All participants were anonymized for personal identity. This study was conducted in accordance with the guidelines of the Declaration of Helsinki and approved by the Institutional Ethics Committee of Xi'an Medical University (approval # XYLS2023081). Informed consent was obtained from all the patients participating in the study.

### 2.10 Immunofluorescent staining for PDIA3

The tissue samples on the slides were dewaxed and rehydrated using a gradient alcohol series, followed by the blocking of endogenous peroxidase through incubation with 3 % hydrogen peroxide. Antigen retrieval was performed using sodium citrate acid buffer (pH 6.0) in a microwave oven. The slides were then incubated overnight at 4 °C with primary rabbit polyclonal anti-PDIA3 antibody (1:300; Proteintech, USA). Subsequently, the slides were incubated with FITC-goat anti-rabbit secondary antibody (Boster Biological Technology, China) at 37 °C for 30 min, followed by washing with PBS three times. The fluorescent signals were captured using a fluorescence microscope (Nikon, Japan), and the staining intensity was quantified using ImageJ software (NIH, USA).

### 2.11 Immunohistochemistry (IHC)

Slides were prepared from resected OSCC and pericarcinomatous tissues. Tissues were dewaxed and rehydrated using a graded alcohol series. Endogenous peroxidase activity was blocked by incubation with 3 % hydrogen peroxide. Antigen retrieval was performed using a sodium citrate buffer (pH 6.0) in a microwave oven. After overnight incubation at 4 °C with rabbit polyclonal anti-PDIA3 primary antibody (1:300, Proteintech, USA), the slides were incubated with HRP-goat anti-rabbit IgG secondary antibody (Boster Biological Technology Co. Ltd, China) at 37 °C for 30 min and washed with PBS three times. The slides were incubated with DAB (Boster Biological Technology Co. Ltd, China), re-stained with hematoxylin, and differentiated with 0.1 % HCl alcohol for 1 s. Finally, the slides were dried by gradient alcohol dehydration, made transparent with xylene, and sealed with neutral gum. The staining intensity was evaluated using ImageJ software (National Institutes of Health, Bethesda, MD, USA).

### 2.12 Wound healing assay

CAL27 and SCC25 cells transfected with siPDIA3 or control siRNA were plated in 6-well plates with cross-marker lines on their backs. When the cells reached 100 % confluence, artificial wounds were created by scratching the cells with a 10-μL pipette tip. Wound healing was observed using an inverted microscope at 0, 12, 24, and 48 h. The rate of wound healing was evaluated using ImageJ software (NIMH, USA). The relative migration distance was calculated using the following formula: percentage of wound closure (%) = 100 × (A − B)/A, where A and B represent the width of cell scratches before and after incubation, respectively.

### 2.13 Correlation between PDIA3 and the PI3K/AKT signaling pathway

The “Limma” package for R software (version 3.6.3) was used to identify the differential expression of PI3K/AKT pathway-related genes in 329 OSCC patients and 32 adjacent normal tissues, and the “ggplot2″ package was used for visualization. The correlation between PDIA3 and the components of the PI3K/AKT signaling pathway was evaluated using Spearman's correlation analysis.

### 2.14 Gene set enrichment analysis (GSEA)

GSEA was performed to explore the potential biological functions and oncogenic pathways of PDIA3. The hallmark gene sets from the Molecular Signatures Database (MsigDB) were used as reference gene sets [15]. The number of permutations in GSEA analysis was 1000, and the gene sets satisfying FDR<0.25 and P < 0.05 were defined as significantly enriched gene sets. The “DESeq2″ and “ClusterProfiler” packages in R were used to conduct the PDIA3 single-gene GSEA analysis.

### 2.15 Association between PDIA3 expression and immune cell infiltration

To determine the immune cell abundance in the OSCC tissues, we performed a single-sample gene set enrichment analysis (ssGSEA) to estimate the fractions of 24 types of immune cells in each sample using the R package ‘GSVA.' The correlation between PDIA3 expression levels and the abundance of each type of immune cell was evaluated using Spearman correlation analysis. The correlations were visualized using a lollipop plot, which illustrates the correlation coefficient of each type of immune cell alongside its corresponding p-value. A p-value <0.05 was considered a significant correlation.

### 2.16 Statistical analysis

Statistical differences were analyzed using either a *t*-test or one-way ANOVA, as appropriate. Data are presented as the mean ± standard deviation (SD) for each group. A P-value of less significance was set at P < 0.05.

## 3 Results

### 3.1 PDIA3 expression is up-regulated in OSCC tissues

**Fig. 1:**
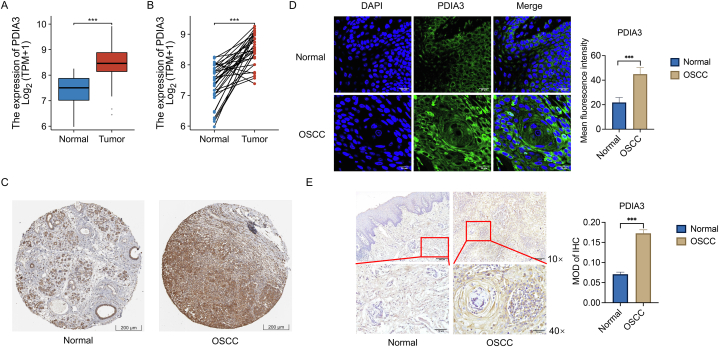
PDIA3 expression is up-regulated in OSCC tissues. (A and B) The mRNA expression of PDIA3 in the normal and OSCC tissues in the OSCC dataset from TCGA. (A) Compare all tumor and normal tissues. (B) Only compare paired tumors and normal tissues. (C) The protein expression of PDIA3 by IHC was retrieved from the HPA database. (D) The immunofluorescent staining of PDIA3 in human OSCC tissues and non-tumorous tissues. Left, representative images; right, quantification of the intensity of the PDIA3 staining. Bar = 20 μm. (E) The IHC staining of PDIA3 in human OSCC tissues and normal tissues. Left, representative IHC images. Upper: bar = 200 μm; lower: bar = 50 μm. Right, quantification of the intensity of the PDIA3 staining. (F) PDIA3 mRNA expression in normal tissues and tumors at different T stages. (G) PDIA3 level in normal tissues and OSCC tumors at different N stages. ***, P < 0.001. A P < 0.05 is significantly different.

We analyzed the mRNA expression of PDIA3 in OSCC tissues and adjacent normal tissues in the OSCC cohort using the TCGA database. The mRNA level of PDIA3 in OSCC tissues was significantly higher than that in the normal tissues (Fig. 1A). Further analysis of paired tumor and normal tissues showed a consistent upregulation of PDIA3 in OSCC tissues (Fig. 1B). Protein expression data from the HPA database also confirmed elevated PDIA3 levels in OSCC tissues compared to those in normal tissues (Fig. 1C). We also evaluated the protein expression of PDIA3 in human OSCC and normal tissues by immunofluorescent staining of PDIA3. The OSCC tissues showed significantly stronger staining of PDIA3 than the normal tissues, indicating that OSCC tissues had higher PDIA3 expression than normal tissues (Fig. 1D). Consistently, the IHC assay also indicated that OSCC tissues had higher expression of PDIA3 than normal tissues (Fig. 1E). Collectively, these findings indicate that PDIA3 is up-regulated in OSCC tissues and suggest a role for PDIA3 in promoting OSCC development. We also evaluated PDIA3 mRNA expression in normal tissues and tumors at different T stages to investigate the potential role of PDIA3 in OSCC progression. Tumors at all T stages showed a significant increase in PDIA3 expression, but no significant difference was observed among different stages of the tumor (Fig. 1F). This suggests that PDIA3 expression remains relatively consistent in OSCC as the tumors grow larger and may not be involved in OSCC progression. Additionally, OSCC at different N stages all exhibited significantly increased expression of PDIA3 compared to normal tissues. However, no significant difference was observed among different N stages of the tumor (Fig. 1G). These findings suggest that PDIA3 may not be implicated in OSCC lymph node metastasis.

## 4 The diagnostic and prognostic value of PDIA3 in OSCC

**Fig. 2:**
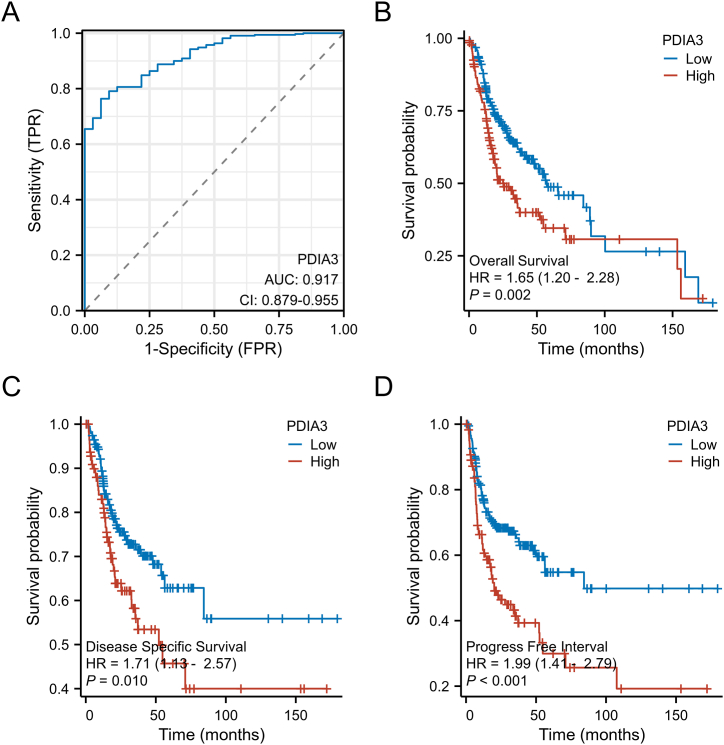
The diagnostic and prognostic value of PDIA3 in OSCC. The PDIA3 expression and clinical data were retrieved from the OSCC dataset in TCGA. (A) The ROC curve for analyzing the value of PDIA3 in the diagnosis of OSCC. (B–D) The Kaplan-Meier method was used to analyze the value of PDIA3 in predicting the prognosis of OSCC patients. The Kaplan-Meier curves for (B) OS, (C) DSS and (D) PFI are shown here. The Logrank test was used to compare the curves. A P < 0.05 is significantly different.

We evaluated the significance of PDIA3 expression in the diagnosis of OSCC by analyzing the TCGA database. The receiver operating characteristic (ROC) analysis revealed that PDIA3 expression exhibited high sensitivity and specificity for OSCC diagnosis and detection (AUC:0.917, CI:0.879–0.955, Fig. 2A), suggesting that PDIA3 expression may serve as a promising diagnostic biomarker for OSCC. We further investigated the potential prognostic value of PDIA3 expression in patients with OSCC using Kaplan-Meier survival analysis. Our results showed that patients with higher PDIA3 expression levels had significantly shorter OS (HR = 1.65, 95 % CI: 1.20–2.28, Log-rank P = 0.002, Fig. 2B), DSS (HR = 1.71, 95 % CI: 1.13–2.57, Log-rank P = 0.010, Fig. 2C), and PFI (HR = 1.99, 95 % CI: 1.41–2.79, Log-rank P < 0.001, Fig. 2D) than those with lower PDIA3 expression levels. These findings suggest that PDIA3 expression is closely associated with poor prognosis in OSCC patients, highlighting its potential as a prognostic biomarker for this disease.

### 4.1 Independent prognostic analysis of PDIA3 and survival

We then performed univariate Cox regression analysis to identify the risk factors for poor prognosis in patients with OSCC. The variables included in this analysis were T and N stages, sex, age, lymphovascular invasion status, previous radiotherapy, and PDIA3 expression. Our analysis revealed that the N2-3 stage, lymphovascular invasion, previous radiotherapy, and high expression of PDIA3 were significantly associated with poor prognosis (Table 1). These factors were included in the subsequent multivariate Cox regression analysis to determine independent prognostic values. Multivariate Cox regression analysis confirmed that the N2-3 stage, lymphovascular invasion, previous radiotherapy, and high expression of PDIA3 were independent risk factors for poor prognosis in patients with OSCC (Table 1). These results suggest that PDIA3 expression, along with other clinical factors, may serve as a biomarker for poor prognosis in patients with OSCC.

**Table 1:** Univariate and multivariate Cox regression analysis of PDIA3 in OSCC.

Variables		Total (N)	Univariate analysis		Multivariate analysis	
			Hazard ratio (95 % CI)	P value	Hazard ratio (95 % CI)	P value
T stage	T1	33	Reference			
	T2	143	1.086 (0.568–2.074)	0.803		
	T3	131	1.461 (0.769–2.773)	0.247		
	T4	179	1.249 (0.665–2.344)	0.490		
N stage	N0	238	Reference			
	N1	80	1.058 (0.728–1.539)	0.768	1.445 (0.850–2.455)	0.174
	N2&N3	161	1.404 (1.038–1.900)	0.028*	1.815 (1.134–2.905)	0.013*
Gender	Female	134	Reference			
	Male	367	0.764 (0.574–1.018)	0.066	0.816 (0.535–1.244)	0.344
Age	≤60	245	Reference			
	>60	256	1.252 (0.956–1.639)	0.102		
Lymphovascular invasion	No	218	Reference			
	Yes	122	1.699 (1.211–2.384)	0.002**	1.817 (1.180–2.798)	0.007**
Radiation therapy	No	153	Reference			
	Yes	287	0.613 (0.452–0.831)	0.002**	0.465 (0.303–0.713)	< 0.001***
PDIA3	Loe	251	Reference			
	High	250	1.519 (1.161–1.986)	0.002**	1.634 (1.102–2.423)	0.015*

OSCC: oral squamous cell carcinoma; PDIA3: protein disulfide isomerase A3; CI: confidence interval. *, P < 0.05; **, P < 0.01, ***, P < 0.001. A P < 0.05 indicates the difference is statistically significant.

### 4.2 Down-regulation of PDIA3 inhibits the cancerous phenotypes of OSCC cells

**Fig. 3:**
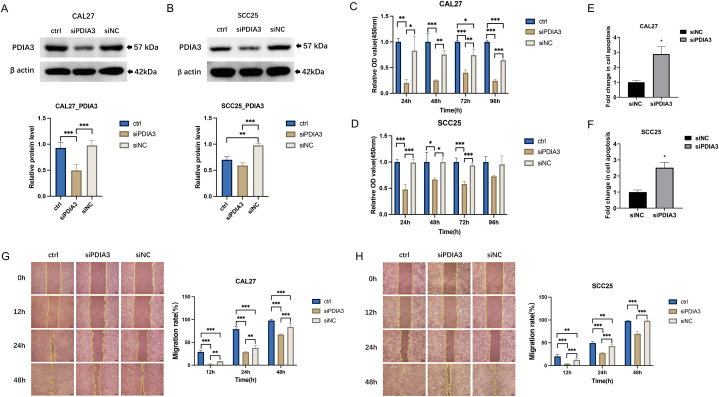
Down-regulation of PDIA3 inhibits the cancerous phenotypes of OSCC cells. The CAL27 and SCC25 cells were transfected with siRNA oligos targeting PDIA3 (designated as siPDIA3). Cells transfected with non-targeting oligos were used as controls (siNC). The untreated cells were also used and designated as ctrl. (A and B) Immunoblotting was performed to measure the protein level of PDIA3 in (A) CAL27 and (B) SCC25 cells. (C and D) The cell viability was measured by CCK8 assay on 24, 48, 72, and 96 h in (C) CAL27 and (D) SCC25 cells transfected with siPDIA3 or non-targeting siRNA. (E and F) The apoptosis of (E) CAL27 and (F) SCC25 cells was measured using an ELISA assay detecting the level of cytosolic nucleosomes. (G and H) The migratory ability of siRNA-transfected (G) CAL27 and (H) SCC25 cells was evaluated using a wound healing assay. Left: representative images; right: quantification of the wound healing rates. Scale bar = 100 μm *, P < 0.05, **, P < 0.01, ***, P < 0.001. A P < 0.05 is significantly different.

The increased expression of PDIA3 in tumor tissues and its association with poor prognosis indicates an oncogenic role of PDIA3 in OSCC. To investigate this, we downregulated PDIA3 expression in two OSCC cell lines, CAL27 and SCC25, by transfecting siRNA oligos targeting PDIA3. Downregulation of PDIA3 was confirmed by immunoblotting (Fig. 3A and B). Knockdown of PDIA3 led to a significant decrease in cell viability (Fig. 3C and D) and an increase in apoptosis (Fig. 3E and F) in both cell lines. Furthermore, the depletion of PDIA3 significantly inhibited the migration ability of OSCC cells (Fig. 3G and H). Collectively, these results indicate that PDIA3 plays an oncogenic role in OSCC.

#### 4.2.1 PDIA3 regulates the PI3K/AKT signaling pathway in OSCC

**Fig. 4:**
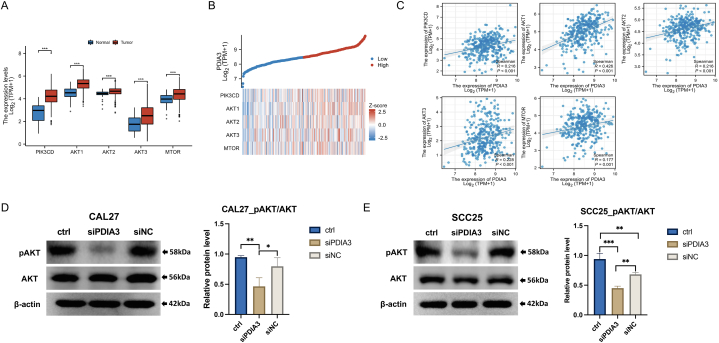
PDIA3 regulates the PI3K/AKT signaling pathway in OSCC. (A) The mRNA expression of PI3KCD, AKT1-3, and mTOR in OSCC and normal tissues. (B) A heatmap showing the expression levels of PI3KCD, AKT1-3, and mTOR across PDIA3^high^ and PDIA3^low^ OSCC tissues. Red: high expression level; blue: low expression level. (C) The correlation between PDIA3 and PI3KCD, AKT1-3, or mTOR was analyzed by Spearman's correlation analysis. (D and E) PDIA3 was silenced by siRNA transfection (siPDIA3). Cells transfected with non-targeting siRNA (siNC) and untreated cells (ctrl) were used as controls. The levels of phosphor-AKT and total AKT were measured by immunoblotting in (D) CAL27 and (E) SCC25 cells. *, P < 0.05, **, P < 0.01, ***, P < 0.001. A P < 0.05 is significantly different.

PDIA3 has been shown to interact with and regulate the activity of AKT and modulate the downstream signaling of the PI3K/AKT pathway, which is a key regulator of cell growth, survival, and metabolism in other types of cancer [[16], [17], [18]]. We found that five components of the PI3K/AKT signaling pathway, PI3KCD, AKT1-3, and mTOR, were up-regulated in OSCC tissues compared with normal tissues (Fig. 4A). Furthermore, their expression was significantly higher in OSCC tissues with high PDIA3 expression than that in OSCC tissues with low PDIA3 expression (Fig. 4B). We then evaluated the correlation between PDIA3 and PI3K/AKT signaling members in OSCC. The results indicated a weak positive correlation between PDIA3 expression and the levels of PI3KCD, AKT1-3, and mTOR, which need to be further examined by experimental studies (Fig. 4C). Additionally, depletion of PDIA3 resulted in a significant downregulation of AKT phosphorylation, whereas the total AKT level remained unchanged (Fig. 4D and E), indicating that PDIA3 may enhance the activity of AKT in OSCC. These findings suggest that PDIA3 may play an oncogenic role by activating the PI3K/AKT signaling pathway in OSCC.

### 4.3 PDIA3 functional enrichment analysis and influence on immune response

**Fig. 5:**
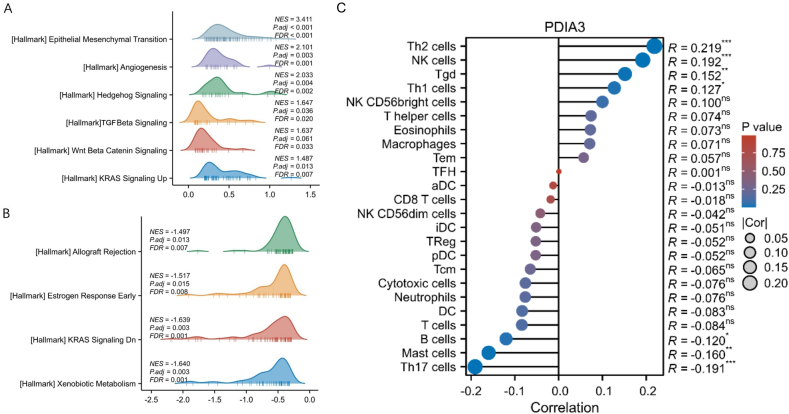
PDIA3 single-gene GSEA functional enrichment analysis. The GSEA for the differential genes related to PDIA3. (A) Enriched functions of the down-regulated genes. (B) Enriched functions of the up-regulated genes. (C) A lollipop plot showing the correlation between PDIA3 expression and immune cell infiltration in OSCC tissues by Spearman analysis. *, P < 0.05, **, P < 0.01, ***, P < 0.001, n.s, not significant. A P < 0.05 is significantly different.

To obtain a broader view of PDIA3 functions in OSCC and investigate potential mechanisms for its role in the disease, we conducted a GSEA for the differential genes related to PDIA3. These genes were identified as those that were differentially expressed between PDIA3^high^ and PDIA3^low^ tumors. The results of the PDIA3 single-gene GSEA analysis showed that the PDIA3-related differentially expressed genes were mainly enriched in several pathways and processes, including epithelial-mesenchymal transition, angiogenesis, Hedgehog signaling pathway, TGF-β signaling pathway, Wnt–catenin signaling pathway, allograft rejection, KRAS signaling pathway, and xenobiotic metabolism (Fig. 5A and B). Furthermore, several studies have highlighted the significant role of PDIA3 in anti-cancer immune responses. It has been shown to either promote or inhibit immune cell infiltration, depending on the tumor type and the specific immune cell involved [19,20]. Nevertheless, the impact of PDIA3 on immune cell infiltration in OSCC has remained largely unexplored. Therefore, we investigated the correlation between PDIA3 expression and infiltration of 24 types of immune cells in OSCC. The results revealed a positive correlation between PDIA3 expression and the infiltration of T-helper 2 (Th2), Natural Killer (NK), Gamma Delta T (Tgd) and Th1 cells, while a negative correlation between PDIA3 and infiltration of B cells, mast cells, and Th17 cells was identified (Fig. 5C). No significant correlation was observed between PDIA3 and infiltration of NK CD56bright, NK CD56dim, T helper cells, eosinophils, macrophages, effector memory T (Tem) cells, T follicular helper (TFH) cells, activated dendritic cells (aDC) cells, CD8^+^ T cells, immature DC (iDC), regulatory T (Treg) cells, plasmacytoid DC (pDC) cells, T central memory (Tcm) cells, cytotoxic cells, neutrophils, DC, and T cells (Fig. 5C). These results suggest that PDIA3 may play a potential role in the immune response in OSCC and warrant further experimental studies in future.

## 5 Discussion

OSCC is a highly prevalent and aggressive cancer of the head and neck region, and despite advancements in treatment modalities, the prognosis for advanced-stage OSCC remains poor, with a five-year survival rate of less than 30 % [21]. However, the current understanding of the formation and development of OSCC is limited. Therefore, there is a critical need to further investigate the molecular drivers of OSCC to improve patient outcomes.

PDIA3 is an important member of the protein disulfide-isomerase (PDI) family and is involved in a variety of biological functions, including receptor stabilization, antigen processing and presentation, and protein degradation [22]. However, the role of PDIA3 in OSCC remains unclear. In this study, we found that PDIA3 was significantly up-regulated in OSCC tissues compared to normal tissues. This result is consistent with that of He et al. who found that PDIA3 was overexpressed in oral squamous cell carcinoma [23]. The expression of PDIA3 is also up-regulated in colon adenocarcinoma [10,24], hepatocellular carcinoma [9], invasive breast carcinoma [25,26], and prostate adenocarcinoma [27]. We further investigated the function of PDIA3 in OSCC cells. Depletion of PDIA3 significantly decreased cell viability, increased apoptosis, and inhibited cell migratory ability in CAL27 and SCC25 cells. These results indicate that PDIA3 plays an oncogenic role in OSCC. This result is consistent with the role of PDIA3 in other types of cancer. For example, Ye et al. showed that the downregulation of PDIA3 inhibits the proliferation and invasion of human acute myeloid leukemia cells [12]. Kondo et al. showed that downregulation of PDIA3 expression inhibited cell proliferation and induced apoptosis through STAT3 signaling in hepatocellular carcinoma [28]. PDIA3 is also related to the development of drug and radiation resistance in cancers. Choe et al. showed that PDIA3 is up-regulated in radioresistant laryngeal cancer cells and potentiates radioresistance of laryngeal cancer cells by increasing STAT3 activity through direct interaction [13]. Song et al. showed that downregulation of PDIA3 inhibits the proliferative, migratory, and invasive capacities of multidrug-resistant gastric cancer cells [29]. Depletion of PDIA3 in colon cancer cells inhibits their proliferation and increases their sensitivity to ionizing radiation and chemotherapeutics [24]. These studies, together with our study, indicate that PDIA3 plays an oncogenic role in human cancers including OSCC. Notably, studies regarding the role of PDIA3 in OSCC are limited. Further cell line-based and animal studies are required to fully understand the role of PDIA3 in OSCC.

One of the main reasons for the poor prognosis of OSCC is late diagnosis, as the five-year survival rate of advanced patients remains less than 30 % [21]. Therefore, the identification of effective diagnostic and prognostic biomarkers is important for improving the outcomes of patients with OSCC. In this study, the ROC curve showed that PDIA3 had high sensitivity and accuracy for the diagnosis of OSCC. We also found that high expression of PDIA3 was closely related to the poor prognosis of patients with OSCC. Univariate and multivariate Cox regression analyses showed that the expression level of PDIA3 was an independent prognostic indicator for OSCC patients. Collectively, these results indicate that PDIA3 can serve as an effective diagnostic and prognostic biomarker for OSCC to further improve patient outcomes. In hepatocellular carcinoma, increased expression of PDIA3 is associated with poor prognosis [9]. PDIA3 is also up-regulated in cervical cancer tissues, and elevated expression of PDIA3 is associated with shorter survival of patients, suggesting that PDIA3 can be used as a marker of poor prognosis in cervical cancer [30]. Together, these currently available studies indicate that PDIA3 is associated with a shorter survival time in cancer patients and may be used as a biomarker for poor prognosis in cancer patients.

In recent years, PDIA3 has been shown to interact with and regulate the activity of the AKT signaling pathway, a key regulator of cell growth, survival, and metabolism in several types of cancer [31]. Given the importance of the PI3K/AKT signaling pathway in cancer, we studied the association between PDIA3 and the AKT pathway in OSCC. We found that three components of AKT signaling, PI3KCD, AKTs, and mTOR, were up-regulated in OSCC tissues, and PDIA3 expression was positively correlated with the levels of PI3KCD, AKTs, and mTOR. Furthermore, depletion of PDIA3 resulted in a significant downregulation of AKT phosphorylation, while the total AKT level remained unchanged. This indicated that PDIA3 may enhance AKT activity in OSCC cells. However, further studies are needed to investigate whether PDIA3 plays a role in cancers by activating the PI3K/AKT signaling pathway. In addition to the AKT pathway, other signaling pathways have also been reported to be involved in PDIA3-mediated biological functions in cancers, such as STAT3 signaling [28], EGFR signaling [32], 1alpha, 25-dihydroxy vitamin D3 [1,25(OH)2D3]-mediated pathway [33] and mTOR signaling [31]. Notably, the correlation between PDIA3 and these signaling pathways in OSCC is unknown. Further studies are needed to investigate the molecular mechanisms underlying the oncogenic role of PDIA3 in OSCC and other types of cancer.

To obtain a broader view of the functions of PDIA3 in cancer cells and to identify more potential mechanisms for the role of PDIA3 in human cancers, we performed GSEA for the differential genes related to PDIA3. GSEA analysis showed that PDIA3 and its related differential genes are involved in a variety of important biological functions closely related to cancer and affect OSCC through the Hedgehog, TGF-β, Wnt-β catenin, and KRAS signaling pathways. Detection or intervention of PDIA3 expression levels in related tissues may become a new target for the early diagnosis and targeted therapy of OSCC. However, the involvement of PDIA3 in these cellular functions and signaling pathways and the contribution of these biological functions to the role of PDIA3 in OSCC require further investigation in future studies. We also discovered that PDIA3 expression had a positive correlation with the infiltration of Th2, NK, Tgd, and Th1 cells and a negative relationship with B cell, mast cell, and Th17 cell infiltration. These results suggest that PDIA3 may be associated with enhanced anti-tumor immune response in OSCC, because Th2, NK, Tgd, and Th1 generally promote the anti-tumor immune responses [[34], [35], [36]]. These results align with some published studies showing PDIA3 is implicated in anti-cancer immune responses [19,20,37]. For example, Zhang et al. reported that PDIA3 correlated with the infiltration of macrophages and T cells and was involved in the suppression of anti-tumor immunity in gliomas [37]. In another study, PDIA3 expression was found to be negatively correlated with infiltration of B cell memory, T cell regulatory, monocytes, and macrophages M2, but positively correlated with NK cell activated and mast cells activated in cervical cancer [30]. PDIA3's impact on immune cell infiltration in cancer is complex and context-dependent. It can promote or inhibit immune cell infiltration in cancers. Notably, the currently available studies mainly focus on the observation that PDIA3 is associated with immune cell infiltration. The mechanism accounting for the phenomenon was still not clear. For example, the primary function of PDIA3 is related to protein folding and quality control in the ER [38]. Whether PDIA3 can influence the immune response by regulating the folding of immune-related protein factors is still unclear. Additionally, the role of PDIA3 in the immune response and immune cell infiltration in OSCC remains largely unknown and needs to be further studied by more experimental studies in the future.

In summary, PDIA3 expression was up-regulated in OSCC tissues compared to that in normal tissues. Downregulation of PDIA3 significantly decreased cell viability, increased apoptosis, and inhibited migratory ability. PDIA3 has high efficiency and accuracy in detecting OSCC and is a good predictor of poor prognosis in patients with OSCC. PDIA3 expression is associated with the expression of the AKT pathway and regulates AKT activity. Functional enrichment analysis indicated that PDIA3 is involved in multiple cancer-related cellular functions and signaling pathways.

## 6 Conclusion

In conclusion, PDIA3 is up-regulated in OSCC, and overexpression of PDIA3 is associated with poor survival of patients. PDIA3 promotes OSCC cell growth, likely through enhancing the activity of the PI3K/AKT signaling pathway. PDIA3 may serve as an effective diagnostic and prognostic biomarker for OSCC. Notably, the role of PDIA3 in OSCC and its underlying mechanisms need to be further investigated in future studies.

## Ethics approval and consent to participate

This study was approved by the Institutional Ethics Committee of Xi'an Medical University (#XYLS2023081). Informed consent was obtained from all the patients in the study.

## Financial support

This project was supported by funds from the Tooth Tissue/Dentition Defect Etiology Prevention, Treatment, and Restoration Innovation Team (#2021TD04) and 10.13039/501100017614Xi'an Medical University College Students' Innovation and Entrepreneurship Training Program Project (#121523031).

## Data availability

No data associated with this study needs to be deposited into a publicly available repository. The analysis presented in this study utilized publicly available databases through bioinformatics tools. All relevant results and findings from the bioinformatics analysis are comprehensively detailed within the manuscript and the accompanying supplemental materials. Therefore, no additional data need to be deposited.

## CRediT authorship contribution statement

**Lin Wang:** Writing – review & editing, Writing – original draft, Validation, Methodology, Investigation, Conceptualization. **Xinxin Wang:** Validation, Investigation, Conceptualization. **Jia Zhang:** Resources. **Jiafeng Duan:** Resources. **Chengfang Tang:** Supervision, Resources, Project administration, Methodology, Conceptualization. **Linmei Zhang:** Investigation. **Hui Zeng:** Investigation. **Hantong Li:** Visualization, Formal analysis, Data curation. **Yuefan Li:** Visualization, Formal analysis, Data curation. **Yan Zhou:** Visualization, Formal analysis, Data curation.

## Declaration of competing interest

The authors declare that they have no known competing financial interests or personal relationships that could have appeared to influence the work reported in this paper.

## References

[1] Kato M.G. (2020). Update on oral and oropharyngeal cancer staging - international perspectives. World journal of otorhinolaryngology - head and neck surgery.

[2] Markopoulos A.K. (2012). Current aspects on oral squamous cell carcinoma. Open Dent. J..

[3] Torre L.A. (2015). Global cancer statistics. CA Cancer J Clin.

[4] de Abreu P.M. (2018). Frequency of HPV in oral cavity squamous cell carcinoma. BMC Cancer.

[5] Kim J.W. (2016). Prognostic value of glucosylceramide synthase and P-glycoprotein expression in oral cavity cancer. Int. J. Clin. Oncol..

[6] Hettinghouse A., Liu R., Liu C.J. (2018). Multifunctional molecule ERp57: from cancer to neurodegenerative diseases. Pharmacol. Ther..

[7] Liu Y. (2019). Upregulation of ERp57 promotes clear cell renal cell carcinoma progression by initiating a STAT3/ILF3 feedback loop. J. Exp. Clin. Cancer Res..

[8] Zhu Y. (2016). Depletion of Dicer promotes epithelial ovarian cancer progression by elevating PDIA3 expression. Tumour Biol.

[9] Takata H. (2016). Increased expression of PDIA3 and its association with cancer cell proliferation and poor prognosis in hepatocellular carcinoma. Oncol. Lett..

[10] Yang Z. (2018). Expression of protein disulfide isomerase A3 precursor in colorectal cancer. OncoTargets Ther..

[11] Staquicini F.I. (2021). Targeting a cell surface vitamin D receptor on tumor-associated macrophages in triple-negative breast cancer. Elife.

[12] Ye Q. (2018). Downregulation of PDIA3 inhibits proliferation and invasion of human acute myeloid leukemia cells. OncoTargets Ther..

[13] Choe M.H. (2015). ERp57 modulates STAT3 activity in radioresistant laryngeal cancer cells and serves as a prognostic marker for laryngeal cancer. Oncotarget.

[14] Uhlen M. (2017). A pathology atlas of the human cancer transcriptome. Science.

[15] Liberzon A. (2015). The Molecular Signatures Database (MSigDB) hallmark gene set collection. Cell Syst.

[16] Wang C. (2020). The role of PDIA3 in myogenesis during muscle regeneration. Exp. Mol. Med..

[17] Ocklenburg T. (2021). *In* oxygen-deprived tumor cells ERp57 provides radioprotection and ensures proliferation via c-Myc, PLK1 and the AKT pathway. Sci. Rep..

[18] Revathidevi S., Munirajan A.K. (2019). Akt in cancer: mediator and more. Semin. Cancer Biol..

[19] Song D. (2021). Insights into the role of ERp57 in cancer. J. Cancer.

[20] Tu Z. (2022). Protein disulfide-isomerase A3 is a robust prognostic biomarker for cancers and predicts the immunotherapy response effectively. Front. Immunol..

[21] Leemans C.R., Braakhuis B.J., Brakenhoff R.H. (2011). The molecular biology of head and neck cancer. Nat. Rev. Cancer.

[22] Mo H.Q. (2020). PDIA3 regulates trophoblast apoptosis and proliferation in preeclampsia via the MDM2/p53 pathway. Reproduction.

[23] He Y. (2016). Largescale transcriptomics analysis suggests over-expression of BGH3, MMP9 and PDIA3 in oral squamous cell carcinoma. PLoS One.

[24] Hussmann M. (2015). Depletion of the thiol oxidoreductase ERp57 in tumor cells inhibits proliferation and increases sensitivity to ionizing radiation and chemotherapeutics. Oncotarget.

[25] Ramos F.S. (2015). PDIA3 and PDIA6 gene expression as an aggressiveness marker in primary ductal breast cancer. Genet. Mol. Res..

[26] Da Costa G.G. (2015). Comparative proteomics of tumor and paired normal breast tissue highlights potential biomarkers in breast cancer. Cancer Genomics Proteomics.

[27] Song D.Y. (2021). Insights into the role of ERp57 in cancer. J. Cancer.

[28] Kondo R. (2019). Downregulation of protein disulfide-isomerase A3 expression inhibits cell proliferation and induces apoptosis through STAT3 signaling in hepatocellular carcinoma. Int. J. Oncol..

[29] Song D. (2021). Silencing of ER-resident oxidoreductase PDIA3 inhibits malignant biological behaviors of multidrug-resistant gastric cancer. Acta Biochim. Biophys. Sin..

[30] Zhang J. (2022). Expression and prognostic significance of PDIA3 in cervical cancer. Int J Genomics.

[31] Ramírez-Rangel I. (2011). Regulation of mTORC1 complex assembly and signaling by GRp58/ERp57. Mol. Cell Biol..

[32] Gaucci E. (2013). The protein ERp57 contributes to EGF receptor signaling and internalization in MDA-MB-468 breast cancer cells. J. Cell. Biochem..

[33] Chen J. (2010). Protein-disulfide isomerase-associated 3 (Pdia3) mediates the membrane response to 1,25-dihydroxyvitamin D3 in osteoblasts. J. Biol. Chem..

[34] Li Y. (2020). The dual roles of human γδ T cells: anti-tumor or tumor-promoting. Front. Immunol..

[35] Waldman A.D., Fritz J.M., Lenardo M.J. (2020). A guide to cancer immunotherapy: from T cell basic science to clinical practice. Nat. Rev. Immunol..

[36] Wolf N.K., Kissiov D.U., Raulet D.H. (2023). Roles of natural killer cells in immunity to cancer, and applications to immunotherapy. Nat. Rev. Immunol..

[37] Zhang H. (2020). PDIA3 correlates with clinical malignant features and immune signature in human gliomas. Aging (Albany NY).

[38] Wang B. (2022). Protein disulfide isomerases (PDIs) negatively regulate ebolavirus structural glycoprotein expression in the endoplasmic reticulum (ER) via the autophagy-lysosomal pathway. Autophagy.

